# Is it time for anti-CD20 discontinuation in multiple sclerosis? Cumulative risk or cumulative uncertainty

**DOI:** 10.3389/fimmu.2026.1917676

**Published:** 2026-10-22

**Authors:** Aurora Zanghì, Anneke van der Walt, Emanuele D’Amico

**Affiliations:** 1 Department of Medical and Surgical Sciences, University of Foggia, Foggia, Italy; 2 Department of Neurology, The Alfred Hospital, Melbourne, VIC, Australia

**Keywords:** anti-CD20 therapy, B-cell depletion, de-escalation, disease-modifying therapy, multiple sclerosis, ocrelizumab, ofatumumab, rituximab

## Abstract

The widespread adoption of anti-CD20 monoclonal antibodies (anti-CD20 mAbs) in multiple sclerosis (MS) has prompted increasing interest in treatment discontinuation and de-escalation strategies. In this perspective, we critically examine current data on outcomes following anti-CD20 discontinuation, including evidence from registries, prospective cohorts, and real-world studies. Available findings suggest that disease control achieved under anti-CD20 therapy is pharmacologically maintained rather than biologically resolved, and that B-cell repopulation following treatment cessation is heterogeneous and not reliably predictive of clinical stability. Reports of disease reactivation after discontinuation and the absence of validated biomarkers to identify patients who may safely stop therapy highlight substantial uncertainty. Current evidence therefore remains insufficient to support routine anti-CD20 discontinuation in MS, while a cautious, individualized approach—potentially incorporating dose-spacing strategies—may better reflect the current state of knowledge.

## Introduction

The therapeutic landscape of multiple sclerosis (MS) has been fundamentally transformed by B-cell-depleting therapies targeting CD20, encompassing both the widespread off-label use of rituximab and the approved agents such as ocrelizumab, ofatumumab, and ublituximab (1–5). These agents have been highly effective in reducing inflammatory disease activity, achieving sustained reductions in relapse rates and magnetic resonance imaging (MRI) lesion activity and burden across both relapsing-remitting MS (RRMS) and primary progressive MS (PPMS) (6).

With their increasing adoption as first-line or early-escalation therapies, a growing proportion of patients with MS are now receiving long-term B-cell-depleting treatment. However, interest in anti-CD20 discontinuation strategies has outpaced the available evidence. The rationale for considering anti-CD20 de-escalation or discontinuation is multifactorial and includes concerns related to cumulative immunosuppression, infection risk, hypogammaglobulinemia, impaired vaccine responses, pregnancy planning, aging, and long-term treatment burden (7, 8, 52). In parallel, increasing interest has emerged regarding the possibility that a subset of patients may maintain disease stability despite treatment reduction or discontinuation. Unlike pulsed immune reconstitution approaches, anti-CD20 therapies primarily rely on sustained peripheral B-cell depletion, followed by variable repopulation kinetics after treatment interruption (9–11). The biological and clinical implications of B-cell reconstitution, particularly regarding the risk of long-term disease reactivation, remain incompletely understood (12, 13).

In this perspective, we review the current evidence regarding anti-CD20 treatment discontinuation and de-escalation strategies in MS, focusing on five key areas: (1) the mechanisms underlying disease control during anti-CD20 therapy, (2) the variability and biological implications of B-cell repopulation kinetics, (3) the risk of disease reactivation after treatment interruption, (4) patient-selection and monitoring considerations, and (5) the balance between cumulative treatment-related risks and the uncertainties associated with treatment withdrawal.

## Literature approach

This Perspective was informed by a targeted narrative review of peer-reviewed literature, complemented by reference-list screening of relevant original studies and reviews and updated through August 2026. Evidence was prioritized when it directly addressed anti-CD20 discontinuation or de-escalation, extended-interval dosing, B-cell reconstitution, cumulative safety, aging, and post-treatment disease reactivation. Particular weight was given to randomized evidence where available, prospective cohorts, large registry studies, and recent comparative real-world analyses. Because the objective was interpretative rather than a formal systematic evidence synthesis, no meta-analysis or formal risk-of-bias assessment was performed.

## Continuous immune suppression versus durable immune reprogramming

A key misconception in discussions of treatment discontinuation is the assumption that disease stability achieved during anti-CD20 therapy necessarily reflects durable disease control beyond treatment cessation. The mechanism of action of anti-CD20 monoclonal antibodies is straightforward: selective depletion of CD20+ B cells through antibody-dependent cellular cytotoxicity, complement-dependent cytotoxicity, and direct apoptosis (14). This depletion is profound but temporary. Upon cessation of therapy, B-cell repopulation invariably occurs, driven by ongoing lymphopoiesis from bone marrow precursors (15).

Crucially, this repopulation process does not recapitulate the immune alterations observed with reconstitution therapies. Prospective studies of rituximab-treated patients suggest that total B-cell counts typically reach the lower limit of the normal range at 12 months post-infusion, with memory (CD27+) B cells recovering by 16 months (16). However, this timeline exhibits considerable inter-individual variability, and the quality of the reconstituted B-cell compartment, particularly the balance between regulatory and pro-inflammatory B-cell subsets—remains incompletely characterized.

More importantly, there is no convincing evidence that B-cell repopulation after discontinuation of anti-CD20 therapy leads to a stable, treatment-free remission. The biological expectation is re-emergence of pathogenic B-cell populations capable of contributing to MS disease activity through multiple mechanisms: antigen presentation to autoreactive T cells, cytokine production, and potentially antibody-mediated pathology (17, 18).

Thus, the concept of “exiting” anti-CD20 therapy with expectation of sustained disease control is, at present, more aspirational than evidence-based. The pharmacological suppression achieved during treatment should not be mistaken for durable disease modification.

The natural history of untreated MS is characterized by spontaneous fluctuations in inflammatory activity, with some patients experiencing prolonged periods of apparent stability even without treatment (19).

Currently, neurofilament light chain (NfL) has emerged as a promising marker of neuroaxonal injury, but its utility in predicting outcomes after treatment discontinuation remains unvalidated (20). Similarly, MRI metrics, including brain volume loss and paramagnetic rim lesions, show promise but lack prospective validation in discontinuation cohorts (21, 52).

In the absence of tools to distinguish pharmacologically suppressed disease from truly quiescent disease, discontinuation remains an uncertain and insufficiently guided intervention. The apparent stability observed during treatment may reflect either durable disease control that could persist after cessation of treatment, or fragile suppression that would immediately deteriorate upon B-cell reconstitution.

The available evidence suggests that this latter scenario may be more common. In a cohort of clinically isolated syndrome (CIS) patients who discontinued platform therapy after ≥5 years of no evidence of disease activity (NEDA-3), 47% experienced disease reactivation within 5 years of stopping (22). This occurred despite prolonged pre-discontinuation stability and suggests that treatment effect, rather than disease resolution, accounted for the observed quiescence in nearly half of patients.

## B-cell repopulation and disease reactivation

The phenomenon of disease rebound—defined as recurrence of disease activity exceeding pre-treatment levels—is well characterized with certain MS therapies, particularly natalizumab and fingolimod (23, 24). Upon discontinuation of these agents, rapid reconstitution of immune surveillance can trigger severe inflammatory activity, occasionally resulting in catastrophic outcomes. With anti-CD20 therapies, classical rebound phenomena are less well documented, but this absence of systematic data should not be interpreted as absence of risk (25). A recent case report describes severe disease reactivation following transitions from fingolimod to ofatumumab, despite washout periods intended to prevent rebound (26). These observations suggest that the timing and context of anti-CD20 discontinuation may critically influence outcomes.

B-cell reconstitution varies substantially among individuals and is influenced by patient-related factors and baseline B-cell burden (27, 28). The clinically relevant question is whether the timing or phenotype of B-cell return predicts loss of disease control. Available studies do not provide a consistent answer: clinical stability may persist despite substantial peripheral repopulation, whereas some ocrelizumab cohorts have reported increased radiological activity during treatment spacing (29–33).

Peripheral B-cell repopulation after treatment reduction may restore components of pathogenic immune activity, but peripheral counts are not a validated surrogate for post-treatment stability (34). Importantly, incomplete depletion during ongoing therapy is biologically distinct from planned repopulation during dose spacing. Revie et al. reported that >10 B cells/µL during ocrelizumab treatment was associated with an almost fourfold higher risk of progression independent of relapse activity (PIRA) compared with adequate depletion (35). This finding supports the biological relevance of the depth of depletion but does not establish a threshold for safe redosing or discontinuation.

These observations argue against using B-cell kinetics as a stand-alone decision rule. Extended-interval dosing studies have documented greater CD19+ repopulation and less complete depletion than standard schedules (36, 37), but the clinical meaning of this difference remains uncertain and appears to vary across cohorts. Patient characteristics also matter, with younger individuals and those with greater inflammatory activity potentially more vulnerable to disease reactivation after treatment reduction (38).

## Extended-interval dosing: a pragmatic intermediate strategy

Evidence on extended-interval dosing (EID) is increasingly reassuring in selected patients. Across ocrelizumab and rituximab cohorts, several studies have reported preserved short-term clinical control despite longer dosing intervals and greater peripheral B-cell repopulation; in one comparative rituximab study, 12-month dosing after initial stability did not significantly increase NEDA-3 failure despite greater B-cell repopulation (29–33). These observations suggest that sustained complete peripheral B-cell depletion may not be necessary for short-term disease control in all patients. However, definitions of EID range from relatively short infusion delays to intervals of 8–12 months or longer, and available studies differ substantially in patient selection, prior inflammatory activity, imaging surveillance, and follow-up. These differences limit direct comparisons and preclude a single evidence-based interval.

The discordance between biological repopulation and clinical outcomes is clinically relevant: greater B-cell return does not consistently translate into breakthrough disease. Ocrelizumab cohorts have likewise reported comparable clinical and MRI outcomes between EID and standard dosing despite less complete B-cell depletion, supporting the view that peripheral repopulation alone should not be interpreted as treatment failure. This is consistent with rituximab data showing that treatment efficacy may outlast substantial peripheral B-cell repopulation (16). Observational de-escalation studies therefore support EID as a feasible monitored strategy in selected stable patients (36, 37). At the same time, long-term non-inferiority to standard dosing for disability outcomes has not been established. In practice, dose spacing may be more defensible after prolonged clinical and MRI stability when cumulative infection, immunoglobulin, or treatment-burden concerns become relevant, whereas greater caution is warranted in younger patients and in those with recent or historically high inflammatory activity (38).

Taken together, EID may reduce cumulative anti-CD20 exposure in selected patients, but the absence of validated intervals, B-cell thresholds, and long-term comparative data means that it should currently be approached as individualized de-risking rather than as an established exit strategy.

## Long-term safety and treatment withdrawal risk

The argument for anti-CD20 de-escalation is primarily driven by concerns over cumulative safety risks. These concerns are valid and merit careful consideration.

Infection risk represents the most frequently cited safety concern. Anti-CD20 therapies reduce B-cell-mediated immune surveillance, potentially increasing susceptibility to bacterial, viral, and opportunistic infections (39). Real-world comparative effectiveness studies revealed that rituximab is associated with higher hospitalization rates (incidence rate ratio 2.29–4.54) and hypogammaglobulinemia risk (hazard ratio 2.72–4.79) compared to ocrelizumab, though both agents carry these risks to varying degrees (40).

Hypogammaglobulinemia could develop in a subset of patients receiving prolonged anti-CD20 therapy, with consequences including increased infection susceptibility and potentially impaired vaccine responses (41). The incidence appears to increase with treatment duration, raising legitimate concerns about indefinite therapy continuation. Nevertheless, available long-term data on ocrelizumab remain reassuring overall. In a pooled analysis of 13 interventional clinical trials including 6155 patients treated for up to 14 years, rates of serious infections remained generally stable over time, with most events resolving without treatment discontinuation. Importantly, treatment duration was not independently associated with an increased risk of serious infections in multivariate analyses (42). Although reductions in immunoglobulin levels were observed with prolonged treatment, immunoglobulin G (IgG) levels remained above the lower limit of normal in the majority of patients after 12 years of therapy, and hypogammaglobulinemia was not consistently associated with a higher risk of serious infections (42).

Vaccine responses are consistently blunted in patients receiving anti-CD20 therapy, as suggested extensively during the COVID-19 pandemic (43–45). The inability to mount robust humoral responses to novel pathogens represents a meaningful quality-of-life consideration and potential long-term health risk.

However, these safety concerns must be weighed against another, less quantifiable risk: the loss of disease control. At present, cumulative safety risks associated with continued anti-CD20 therapy are partially characterized and can be monitored through routine laboratory surveillance and clinical vigilance. Hypogammaglobulinemia can be detected through immunoglobulin level monitoring; infection risk can be mitigated through prophylactic strategies and patient education; vaccine timing can be optimized around treatment schedules (41).

By contrast, the risks associated with discontinuation are largely undefined. We lack reliable tools to predict which patients will experience disease reactivation, when such reactivation will occur, or how severe it will be. We cannot identify patients who have achieved spontaneous disease remission versus those whose stability is entirely treatment dependent.

Choosing to stop therapy, therefore, replaces a known, monitorable risk profile with an unknown, potentially irreversible one. An inflammatory relapse, particularly with pyramidal involvement, may result in permanent disability that cannot be reversed, even with prompt treatment reinitiation (46). The long-term cost of a single poorly timed discontinuation may substantially outweigh the incremental risks of continued therapy.

This asymmetry argues against framing de-escalation as a binary continue-or-stop decision. Standard dosing, EID, switching to another disease-modifying therapy, and treatment-free discontinuation represent different degrees of treatment reduction and uncertainty. The relevant clinical question is not simply whether anti-CD20 therapy can be stopped, but whether treatment intensity can be reduced without unacceptably increasing the risk of renewed inflammatory activity. Age, previous inflammatory burden, duration of stability, treatment-related safety concerns, and the feasibility of close monitoring should therefore be considered together.

Recent registry-based evidence has provided important insights into the consequences of ocrelizumab discontinuation in clinical practice. In a propensity score-matched analysis from the MSBase cohort, including patients who discontinued ocrelizumab because of safety concerns and matched continuers, treatment interruption was associated with significantly higher annualized relapse rates (ARR 0.08 vs 0.038; p=0.040) (47). Although differences in time to first relapse did not reach statistical significance, discontinuation was also associated with trends toward increased disability progression, including higher risks of 24-week confirmed disability worsening (CDW) and progression independent of relapse activity (PIRA) (47). Overall, these findings suggest that maintaining therapeutic intensity may be important for preserving long-term disease control.

Additional evidence supports the role of anti-CD20 therapies in preventing rebound inflammatory activity following discontinuation of other high-efficacy treatments. In a cohort of patients transitioning from natalizumab to anti-CD20 therapies, rituximab, ocrelizumab, and ofatumumab were all associated with effective suppression of rebound disease activity (48). Rituximab significantly reduced ARR, although modest disability progression persisted, largely driven by PIRA. Importantly, no rebound disease activity was detected across anti-CD20 groups, and survival analyses showed no significant differences in time to disease activity between therapies, supporting a broadly comparable effectiveness profile after natalizumab withdrawal (48).

In progressive and late-onset MS populations, real-world data from a multicenter Italian secondary progressive multiple sclerosis (SPMS) cohort suggest that siponimod is often used after ocrelizumab discontinuation because of cumulative safety concerns; however, a substantial proportion of patients subsequently resumed anti-CD20 therapy, most commonly ocrelizumab, because of persistent disease activity or insufficient disease control after siponimod withdrawal (49).

In late-onset MS populations, evidence comparing anti-CD20 therapies with sphingosine-1-phosphate receptor modulators remains limited, with ongoing real-world registry analyses expected to better define their relative effectiveness and safety profiles (50).

Perhaps the most critical gap in the discontinuation discourse is the absence of validated criteria to identify patients who might safely stop anti-CD20 therapy. Various factors have been proposed as potential predictors of favorable post-discontinuation outcomes: older age, prolonged disease duration, extended period of NEDA status, low baseline disability, and absence of aggressive early disease course (51–53).

Age emerges as the most consistent predictor in available studies. Among CIS patients who discontinued disease-modifying therapy (DMT) after ≥5 years of NEDA-3, those aged >45 years experienced only 13% disease reactivation compared to 54% in patients <45 years (p<0.001) (22). This age effect likely reflects the natural history of MS, in which inflammatory activity tends to decrease with advancing age, partially independent of treatment (54).

However, age alone is insufficient for clinical decision-making. Among patients >45 years with prolonged NEDA status, 13% still experienced disease reactivation—a non-trivial risk that may be unacceptable for many patients and clinicians (22). Moreover, this threshold has not been prospectively validated, and its applicability to anti-CD20 therapies specifically remains uncertain.

More recent high-efficacy treatment data suggest that age interacts with treatment class rather than functioning as a universal stopping threshold. In patients aged 50 years or older with nonactive MS, discontinuation of high-efficacy therapy increased relapse risk overall, whereas no significant excess risk was observed in the anti-CD20 subgroup; importantly, confidence intervals were wide and follow-up remained limited (53). This distinction argues against extrapolating the rebound profile of immune-trafficking therapies to B-cell-depleting agents.

Disease phenotype and stage add a further layer. In early or historically highly inflammatory RRMS, treatment-induced NEDA may provide limited reassurance because pretreatment inflammatory biology remains relevant. In later stable disease, particularly after age 50, the expected inflammatory benefit of continuous treatment may decline while infection and hypogammaglobulinemia become increasingly salient. In PPMS or SPMS, however, low relapse or MRI activity cannot be equated with biological inactivity because disability may continue to accumulate independently of focal inflammatory events; evidence specifically supporting anti-CD20 withdrawal in progressive MS remains sparse (52–54).

NEDA status is frequently invoked as a prerequisite for consideration of discontinuation. The concept is appealing: patients with no relapses, no disability progression, and no MRI activity for extended periods might reasonably be considered to have inactive disease. However, NEDA status during treatment does not distinguish between treatment-suppressed disease and spontaneously inactive disease.

The absence of validated selection criteria means that discontinuation decisions remain largely empirical. In a disease as heterogeneous as MS, a uniform exit strategy is unlikely to be appropriate. Yet without validated stratification tools, premature discontinuation strategies may expose some patients to an avoidable risk of disease reactivation.

Treatment context should also be distinguished. EID reduces cumulative exposure while retaining the same therapeutic mechanism; switching to another disease-modifying therapy preserves pharmacological coverage but changes mechanism; complete discontinuation creates a treatment-free period. These strategies are therefore not interchangeable, and outcomes after switching from another high-efficacy therapy to anti-CD20 treatment should not be extrapolated to outcomes after anti-CD20 withdrawal itself (48).

Beyond efficacy, accumulating real-world data indicate that many safety concerns associated with anti-CD20 therapies can be managed without treatment discontinuation. This concept is reinforced by the multicenter CONFIDENCE cohort, which included more than 2,000 patients with relapsing multiple sclerosis (RMS) and PPMS treated with ocrelizumab (55, 56). Treatment persistence remained remarkably high, with approximately 92% of patients continuing therapy at 24 months, while discontinuations occurred in fewer than 5% of cases and were only rarely driven by safety concerns or lack of efficacy. These findings support a favorable benefit–risk balance in routine clinical practice and suggest that sustained disease control under anti-CD20 therapy is closely linked to treatment persistence rather than durable immune reprogramming (56).

Importantly, the safety profile observed in real-world cohorts has remained consistent with that reported in pivotal randomized trials of ocrelizumab (4, 57). Infections represented the most common adverse events, but serious infections remained relatively infrequent, supporting the overall manageability of treatment-related risks. Long-term pooled analyses extending to approximately 14 years have shown that serious-infection rates remained generally stable over time (42). From a clinical perspective, these data suggest that, in the absence of severe or uncontrolled adverse events, both clinicians and patients generally favor treatment continuation because of concerns regarding disease reactivation after therapy interruption, particularly given the lack of validated biomarkers capable of identifying patients who may safely discontinue therapy.

Collectively, current evidence does not support routine de-escalation or discontinuation strategies for most patients receiving anti-CD20 therapies. Instead, continuous treatment appears to represent the most reliable approach for maintaining disease control, while safety risks seem largely predictable, monitorable, and manageable through structured clinical surveillance and individualized risk mitigation strategies.

Health-economic considerations should be integrated into the same clinical decision framework rather than treated as an isolated argument for treatment reduction. Recent systematic evidence suggests that anti-CD20 therapies are generally cost-effective compared with platform DMTs, whereas comparisons with other high-efficacy therapies are more heterogeneous and strongly dependent on healthcare setting, drug acquisition costs, and modeling assumptions (58). Potential savings from less frequent dosing or treatment cessation must therefore be weighed against the costs of additional clinical and MRI surveillance, rescue treatment, relapse-related care, and potentially irreversible disability. No economic analysis has yet established the superiority of standard dosing, EID, switching, or complete discontinuation as competing anti-CD20 strategies.

## What evidence is missing

Despite the growing clinical interest in anti-CD20 discontinuation strategies, the current evidence base remains limited and fragmented. Several key knowledge gaps continue to hinder evidence-based clinical decision-making. Important evidence gaps also include the lack of validated biomarkers capable of distinguishing treatment-suppressed from truly inactive disease, and the absence of comparative studies evaluating continuation, extended-interval dosing, and complete discontinuation strategies.

Important uncertainties also persist regarding re-escalation strategies after disease reactivation. It remains unknown whether treatment reinitiation fully restores prior disease control or whether discontinuation-associated inflammatory activity may result in irreversible disability accumulation or altered long-term disease trajectory. Finally, subgroup-specific evidence is critically needed. Patients with highly inflammatory early disease subsequently stabilized under anti-CD20 therapy, progressive phenotypes, older individuals, and patients with comorbidities may exhibit substantially different discontinuation risks and therefore require dedicated investigation. Until these evidence gaps are addressed through prospective studies, anti-CD20 discontinuation remains insufficiently supported by robust clinical and biological evidence.

## Practical recommendations for treatment discontinuation

In patients in whom anti-CD20 discontinuation is being considered—whether because of safety concerns, pregnancy planning, aging, comorbidities, or patient preference—a cautious and individualized approach is warranted. Prolonged disease stability should probably represent a minimum prerequisite before treatment interruption is considered. Although no validated threshold exists, most available observational studies suggesting lower reactivation risk involved patients with several years of sustained NEDA status prior to discontinuation.

Careful post-discontinuation monitoring is essential. Clinical follow-up, serial MRI surveillance, and potentially biomarker assessment may help identify early evidence of disease reactivation, although no standardized monitoring protocol has yet been established. Given the possibility of delayed inflammatory recurrence, surveillance should likely extend beyond the first year after treatment interruption. Clear re-escalation strategies should also be defined prospectively. In the absence of validated predictive tools, a low threshold for treatment reinitiation may be appropriate if clinical relapses, new MRI activity, or evidence of disability progression emerge following discontinuation.

For selected patients, EID may represent a pragmatic intermediate strategy before complete withdrawal. Current observational evidence suggests that dose spacing can preserve clinical and radiological control in many stable patients even when peripheral B-cell repopulation occurs. The most defensible candidates are patients with prolonged clinical and MRI stability in whom recurrent infections, declining immunoglobulin levels, comorbidity, or cumulative treatment burden favor reduced exposure; greater caution is warranted in younger patients and in those with recent clinical or MRI activity or a historically highly inflammatory disease course. Monitoring should include clinical assessment, MRI surveillance, and relevant laboratory measures according to the individual risk profile, but no evidence currently supports a universal monitoring frequency or B-cell threshold. A clinical relapse, unequivocal new MRI activity, or sustained disability worsening should prompt reassessment of the extended schedule and consideration of return to standard dosing or another active treatment strategy. Key evidence, clinical implications, and unresolved uncertainties relevant to anti-CD20 de-escalation and discontinuation are summarized in **Table 1**.

**Table 1:** Evidence informing anti-CD20 de-escalation and discontinuation in multiple sclerosis.

Strategy/domain	Quantitative evidence and clinical interpretation	Clinical context/main uncertainty
Complete discontinuation	MSBase data showed a higher ARR after ocrelizumab discontinuation than continuation (0.08 vs 0.038; p=0.040), with non-significant trends toward higher 24-week CDW and PIRA (47).	Greatest residual uncertainty. Consider only selected circumstances; no validated selection criteria or established timing of delayed reactivation.
Extended-interval dosing/B-cell repopulation	In a 6- vs 12-month rituximab comparison (n=608), 12-month dosing did not significantly increase NEDA-3 failure (ITT HR 1.60, 95% CI 0.69-3.68) despite greater B-cell repopulation (30). In a prospective extended-RTX cohort, ARR was 0.03 before and 0.04 after interval extension (p=0.51), with no MRI lesion accrual in 97% of patients (16).	Best-supported in patients stable before dose spacing; greater caution with recent or historically high inflammatory activity. No validated dosing interval, CD19+ threshold, or long-term non-inferiority data.
Age and disease stage	After ≥5 years of NEDA-3, disease reactivation after DMT discontinuation was 13% in patients >45 years versus 54% in those <45 years (p<0.001) (22); anti-CD20-specific and progressive-MS evidence remains limited (52, 53).	Older age and prolonged stability may lower inflammatory risk but do not eliminate it. No validated age cut-off; applicability differs across RRMS, PPMS, SPMS, and treatment contexts.
Cumulative safety	Anti-CD20 treatment is associated with infection, hypogammaglobulinemia, and attenuated vaccine responses (39–45). In pooled data from 6,155 ocrelizumab-treated patients followed for up to 14 years, serious-infection rates remained generally stable over time (42).	Cumulative safety burden may favor de-escalation when clinically meaningful; risk varies with disability, comorbidities, immunoglobulin levels, prior therapy, and treatment duration.
Health economics	Anti-CD20 therapies are generally cost-effective versus platform DMTs, whereas comparisons with other high-efficacy therapies remain heterogeneous and context-dependent (58).	Economic considerations should complement, not replace, clinical risk stratification; direct comparisons of standard dosing, EID, switching, and complete discontinuation are lacking.

References correspond to the numbering in the main manuscript. ARR, annualized relapse rate; CDW, confirmed disability worsening; DMT, disease-modifying therapy; EID, extended-interval dosing; MRI, magnetic resonance imaging; PIRA, progression independent of relapse activity; PPMS, primary progressive multiple sclerosis; RRMS, relapsing-remitting multiple sclerosis; SPMS, secondary progressive multiple sclerosis; CI, confidence interval; HR, hazard ratio; ITT, intention-to-treat; NEDA-3, no evidence of disease activity-3; RTX, rituximab.

Importantly, patients should be informed that anti-CD20 discontinuation is not currently supported by high-level evidence and that the biological and clinical consequences of treatment withdrawal remain incompletely understood. Shared decision-making therefore remains central to any de-escalation strategy.

## Conclusion

The increasing long-term use of B-cell-depleting therapies in MS has intensified concerns regarding cumulative immunosuppression, infection risk, and treatment burden. These concerns are clinically relevant and may justify treatment discontinuation in selected cases, including pregnancy planning, adverse events, aging, or patient preference. However, current evidence does not support routine discontinuation of anti-CD20 therapy in patients with stable MS. Disease stability achieved on therapy likely reflects ongoing pharmacological suppression rather than durable immune reprogramming, and reliable biomarkers to identify patients who may safely discontinue treatment remain unavailable.

Further prospective studies are needed to directly compare continuation, EID, switching, and discontinuation strategies and to validate predictive biomarkers. From an evidence perspective, three levels should currently be distinguished. First, standard-interval anti-CD20 therapy retains the most established long-term evidence base and remains the reference strategy when efficacy and safety are acceptable. Second, EID is supported by a growing, predominantly observational evidence base and represents a reasonable individualized de-escalation option in selected stable patients, including when peripheral B-cell repopulation occurs without renewed disease activity. Third, complete treatment-free discontinuation remains supported by substantially more limited evidence and should therefore be reserved for carefully selected circumstances with close follow-up.
